# A clinical decision aid to discern patients without and with midfacial and mandibular fractures that require treatment (the REDUCTION-II study): a prospective multicentre cohort study

**DOI:** 10.1007/s00068-022-01892-4

**Published:** 2022-02-24

**Authors:** Romke Rozema, Mostafa El Moumni, Gysbert T. de Vries, Frederik K. L. Spijkervet, René Verbeek, Jurrijn Y. J. Kleinbergen, Bas W. J. Bens, Michiel H. J. Doff, Baucke van Minnen, The REDUCTION study group

**Affiliations:** 1 Department of Oral and Maxillofacial Surgery, University Medical Center Groningen, University of Groningen, Hanzeplein 1, 9700 RB Groningen, The Netherlands; 2 Department of Trauma Surgery, University Medical Center Groningen, University of Groningen, Groningen, The Netherlands; 3 Department of Emergency Medicine, Isala Hospital, Zwolle, The Netherlands; 4 Department of Emergency Medicine, Nij Smellinghe Hospital, Drachten, The Netherlands; 5 Department of Oral and Maxillofacial Surgery, Isala Hospital, Zwolle, The Netherlands; 6 Department of Oral and Maxillofacial Surgery, Nij Smellinghe Hospital, Drachten, The Netherlands; 7 Department of Emergency Medicine, University Medical Center Groningen, University of Groningen, Groningen, The Netherlands

**Keywords:** Maxillofacial fractures, Physical examination findings, Diagnostic accuracy, Clinical decision aid, Treatment, Emergency service, hospital, Craniocerebral trauma, Signs and symptoms, Therapeutics

## Abstract

**Purpose:**

To assess the diagnostic accuracy of physical examination findings and to construct clinical decision aids to discern emergency department patients without and with midfacial and mandibular fractures that require treatment.

**Methods:**

A prospective multicentre cohort study was conducted in four hospitals in the Netherlands. Consecutive maxillofacial trauma patients were included whereupon each patient underwent a standardized physical examination consisting of 15 and 14 findings for midfacial and mandibular trauma, respectively. The primary outcome was the decision whether to treat during the emergency department stay or within 24 h of admission. The diagnostic accuracy was calculated for the individual physical examination findings and ensuing clinical decision aids with the focus being on detecting midfacial and mandibular fractures that require active treatment.

**Results:**

A total of 766 midfacial trauma patients were identified of whom 339 (44.3%) had midfacial fractures. Of those, 74 (21.8%) required active treatment. A total of 280 mandibular trauma patients were identified of whom 66 (23.6%) had mandibular fractures. Of those, 37 (56.0%) required active treatment. The decision aid for midfacial trauma consisting of facial depression, epistaxis, ocular movement limitation, palpable step-off, objective malocclusion and tooth mobility or avulsion had a sensitivity of 97.3 (90.7–99.3), a specificity of 38.6 (35.0–42.3), and a negative predictive value of 99.3 (97.3–99.8). The decision aid for mandibular trauma consisting of mouth opening limitation, jaw movement pain, objective malocclusion and tooth mobility or avulsion resulted in a sensitivity of 100.0 (90.6–100.0), a specificity of 39.1 (33.2–45.4), and a negative predictive value of 100.0 (96.1–100.0).

**Conclusion:**

The clinical decision aids successfully identified midfacial and mandibular trauma patients requiring active fracture treatment and so may be useful in preventing unnecessary radiological procedures in the future.

**Trial Registration:**

The study was registered at ClinicalTrials.gov with the identifier NCT03314480.

**Supplementary Information:**

The online version contains supplementary material available at 10.1007/s00068-022-01892-4.

## Introduction

Midfacial and mandibular fractures are frequently found in trauma patients in the emergency department [1, 2]. Missing these fractures may have major long-term morphological, functional and esthetic consequences. Upon entering the emergency department, each patient should be subjected to a structured assessment of the maxillofacial region and the observed findings should be used to identify which maxillofacial patients may have midfacial or mandibular fractures [1].

Although various studies have focused on how physical examination findings can be used to predict midfacial and mandibular fractures [3–13] and to stratify patients at risk of fractures and subsequently requiring radiological imaging of the maxillofacial region, studies on identifying patients that require treatment are limited [10, 11]. In today’s emergency department landscape, the primary assessment of trauma patients is mostly performed by emergency physicians and specialized trauma surgeons and, if maxillofacial fractures are diagnosed, an oral and maxillofacial surgeon is consulted to assess the need for active treatment. Therefore, early recognition of any fractures by all these health care professionals from the physical examination findings is required to deliver more accurate patient management. Moreover, it allows prioritization of other injuries and optimization of emergency department workflows. A clinical decision aid using physical examination findings could be used as a fast bedside strategy to single out patients with maxillofacial fractures that require treatment but, to date, no such clinical decision aid has been published.

Hence, this prospective multicenter REDUCTION-II study (*RED*ucing *U*nnecessary *C*omputed *T*omography *I*n *M*axill*O*facial I*N*jury) was initiated with a twofold aim. First, to identify the diagnostic accuracy of physical examination findings in identifying midfacial and mandibular fractures that require treatment. Second, the construct a clinical decision aid with the focus being on successfully ruling out patients with midfacial and mandibular fractures requiring treatment in emergency department patients.

## Materials and methods

### Study design

A prospective observational cohort study was conducted of all emergency department patients suspected of midfacial and mandibular trauma between the period of May 2018 and October 2019. The Medical Ethical Committee of the University Medical Center Groningen confirmed that the Medical Research Involving Human Subjects Act did not apply and local feasibility was approved for the participating hospitals. The study was performed in compliance with the Declaration of Helsinki and according to the FEDERA (Foundation Federation of Dutch Medical Scientific Societies) code of conduct. The study was registered at ClinicalTrials.gov (NCT03314480) and reported according to the STARD guidelines (Standards for Reporting of Diagnostic Accuracy Studies) and Methodologic Standards for Interpreting Clinical Decision Rules in Emergency Medicine [14, 15].

### Inclusion and exclusion criteria

All consecutive emergency department patients presenting with midfacial or mandibular trauma at the University Medical Center Groningen (level I), Isala hospital Zwolle (Level I), Isala Diaconessenhuis hospital Meppel (level III) and Nij Smellinghe hospital Drachten (level III) were included. Patients younger than 18 years of age and patients admitted for a second time for maxillofacial trauma within the period of inclusion were excluded. Patients were also excluded if the initial assessment was performed in another hospital or access to medical records was declined.

### Physical examination and radiological imaging

All eligible patients received a standardized full physical examination of the midfacial or mandibular region. The physical examination consisted of 15 findings for midfacial trauma, and 14 findings for mandibular trauma. The findings were consulted during the primary or secondary assessment of the patient, and standardized for all the included patients according to a tripartite strategy consisting of an individual hands-on instruction, online educational tool and bedside use of a pocket card. The findings were scored as absent, present or not assessable. Further details regarding the process of standardization were provided previously by our research group. Patients suspected of midfacial fractures were examined using Computed Tomography (CT) or Cone Beam Computed Tomography (CBCT). Midfacial fractures were defined as any fracture of the frontal sinus, orbital rim and walls, maxillary sinus, zygomaticomaxillary complex, nasoorbitoethmoid (NOE) complex, nasal bone, Le Fort I, II, III complex, and maxillary dentoalveolar complex. Patients suspected of mandibular fractures were diagnosed with CT, CBCT or orthopantomography (OPT). Mandibular fractures were defined as any fracture of the symphyseal or parasymphyseal area, corpus, angle, ramus, coronoid process, condylar process and dentoalveolar complex. Radiological interpretation was performed without knowledge of the radiological imaging outcome and the classification of fractures was performed by a board-certified oral and maxillofacial surgeon (BvM).

### Treatment and outcome measures

The primary outcome was the decision for treatment of midfacial or mandibular fractures as intended during the emergency department stay or within 24 h of admission. The decision of treatment was determined by a consultant oral and maxillofacial surgeon or otorhinolaryngologist. Decisions were made according to the usual care in agreement with the treatment protocols of the Dutch Society of Oral and Maxillofacial Surgery (NVMKA) or Dutch Association of Otorhinolaryngology and Head & Neck Surgery (NVKNO) as consulted within the period of inclusion. The decision of fracture treatment was assigned to either a conservative or active intend. Conservative treatment included adequate analgesics, avoidance of nose blowing or holding the nose when sneezing, a soft non-chewing diet, and watchful observation. Active treatment was divided into closed or open treatment. Closed treatment included reduction of nasal fractures under local anesthesia, nasal packing, intermaxillary fixation, rigid and flexible splinting or appliances for dental injury. Open treatment included any surgical intervention in which the patient underwent open reduction and internal fixation in an operation theater.

Secondary outcomes included the presence of skull fractures and dental injury. Skull fractures were defined as any fracture of the skull base, frontal, temporal, parietal or occipital bone diagnosed with a CT. Dental injury was defined as any clinical observed avulsion, luxation or fracture of the maxillary or mandibular teeth.

### Statistical analyses

The Statistical Package for the Social Sciences was used for the data analyses (IBM Corp. Released 2015. IBM SPSS Statistics for Windows, Version 23.0. Armonk, NY: IBM Corp.). Fracture outcomes were presented as frequencies and percentages. The individual physical examination findings were presented as the proportion of patients diagnosed with a fracture, and patients diagnosed with any fracture requiring active treatment. For the subtypes of fractures, the physical examination findings were presented as the proportion of total diagnosed midfacial and mandibular fractures. The diagnostic accuracy was calculated for each individual physical examination finding.

Principle component analysis (PCA) was used to construct clinical decision aids consisting of physical examination findings, with the focus being on ruling out patients that require active treatment for midfacial or mandibular fractures. The PCA analysis was performed with subsequent promax rotation and Kaiser normalization and used to identify the underlying structure of the physical examination findings. The Bartlett’s test of sphericity and the Kaiser–Meyer–Olkin measure of sampling adequacy were conducted to test whether the variables were uncorrelated in the correlation matrix and factors with Eigenvalues greater than one were initially retained for the analysis.

The physical examination findings selected to construct the clinical decision aids were based on a combination of factor loadings and the clinical considerations of findings related to fractures that require active treatment by two board-certified oral and maxillofacial surgeons (MD and BvM). Objective malocclusion and tooth mobility or avulsion were intentionally included in both clinical decision aids because they are essential findings for each patient suspected of midfacial and mandibular fractures requiring treatment. The contingency tables for the clinical decision aids were constructed with absent findings being recorded as ‘negative’ whereas present, not testable and missing findings were recorded as ‘positive’.

Regarding the outcome of interest, a ‘positive outcome’ was defined as a patient whose fractures underwent active treatment (e.g., closed or open treatment), and a ‘negative outcome’ was defined as patients whose fractures were treated conservatively or patients who had been diagnosed as not having a fracture. The diagnostic accuracy and corresponding 95 percent confidence interval outcomes included: prevalence, pre-test probability, sensitivity, specificity, positive predictive value (PPV), negative predictive value (NPV), positive likelihood ratio (LR+) and negative likelihood ratio (LR−).

## Results

### Patient characteristics

**Fig. 1:**
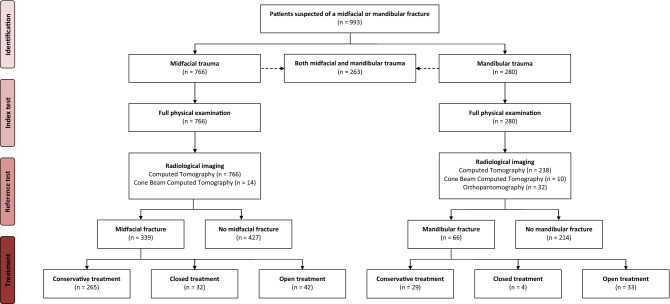
Flowchart of study patients

A total of 993 patients were eligible for inclusion. Among this population, 766 patients had suffered a midfacial trauma and 280 patients had suffered a mandibular trauma. From the total population, 263 patients were identified with both a midfacial and mandibular trauma. Skull fractures were observed in 51 (5.1%) patients (Fig. 1). Dental injury of the maxillary teeth was observed in 83 (8.4%) patients, and dental injury of the mandibular teeth was found in 28 (2.8%) patients.

### Treatment of midfacial fractures

Midfacial fractures were diagnosed in 44.3% (*n* = 339) of the patients. Zygomaticomaxillary complex fractures (*n* = 134), nasal bone fractures (*n* = 126) and orbital rim and wall fractures (*n* = 96) were the most common (Table 1). Among those diagnosed with a midfacial fracture, 265 (78.2%) patients were treated conservatively, 32 (9.7%) received closed treatment and 42 (12.4%) received open treatment. The treatment outcomes of the midfacial fracture subtypes are presented in Table 1. Conservative treatment occurred most commonly for patients suffering fractures of the frontal sinus (64.0%), orbital rim and walls (84.4%), maxillary sinus (86.7%), zygomaticomaxillary complex (79.9%), nasoorbitoethmoid complex (70.6%) and the nasal bone (70.6%). Le Fort type fractures were generally treated surgically.

**Table 1:** Fracture outcomes

Fracture type	Total (*n*)	Conservative treatment (*n* (%))	Closed treatment (*n* (%))	Surgical treatment (*n* (%))
Midface fractures	339	265 (78.2)	32 (9.4)	42 (12.4)
Frontal sinus	25	16 (64.0)	0 (0.0)	9 (36.0)
Orbital rim and walls	96	81 (84.4)	4 (4.2)	11 (11.5)
Maxillary sinus	30	26 (86.7)	2 (6.7)	2 (6.7)
Zygomaticomaxillary complex	134	107 (79.9)	0 (0.0)	27 (20.1)
Nasoorbitoethmoid complex	17	12 (70.6)	1 (5.9)	4 (23.5)
Nasal bone	126	89 (70.6)	24 (19.0)	13 (10.3)
Le Fort I	9	4 (44.4)	1 (11.1)	4 (44.4)
Le Fort II	8	2 (25.0)	0 (0.0)	6 (75.0)
Le Fort III	6	3 (50.0)	0 (0.0)	3 (50.0)
Dentoalveolar complex	15	6 (40.0)	8 (53.3)	1 (6.7)
Mandible fractures	66	29 (43.9)	4 (6.1)	33 (50.0)
Symphyseal or parasymphyseal	24	6 (25.0)	0 (0.0)	18 (75.0)
Corpus	17	5 (29.4)	2 (11.8)	10 (58.8)
Angular	8	0 (0.0)	0 (0.0)	8 (100.0)
Ramus	7	4 (57.1)	0 (0.0)	3 (42.9)
Coronoid	4	1 (25.0)	0 (0.0)	3 (75.0)
Condylar process	44	21 (47.7)	4 (9.1)	19 (43.2)
Dentoalveolar complex	1	0 (0.0)	0 (0.0)	1 (100.0)

### Findings related to midfacial fractures requiring treatment

Facial depression (93.9%), ocular movement limitation (92.3%), infra-orbital nerve paresthesia (80.6%) and palpable step-off (91.2%) were the physical examination findings most often associated with the presence of a midfacial fracture (Table 2). The physical examination findings that were often seen with midfacial fractures requiring active treatment were facial depression (46.9%), palpable step-off (41.2%), objective malocclusion (39.1%), tooth mobility or luxation (35.4%) and ocular movement limitation (30.8%). The physical examination finding outcomes midfacial fracture subtypes requiring surgical treatment are presented in Table 2.

**Table 2:** Physical examination findings associated with midfacial fractures requiring active treatment

Physical examination findings	Total observed	Midfacial fractures (*n* (%)) ^1^		Midfacial fracture subtypes requiring active treatment (*n* (%)) ^2^									
		Fractures identified	Fractures requiring active treatment	Frontal sinus	Orbital rim and wall	Maxillary sinus	Zygomaticomaxillary complex	Nasoorbitoethmoid complex	Nasal bone	Le Fort I	Le Fort II	Le Fort III	Dentoalveolar complex
Swelling	621	294 (47.3)	63 (10.1)	9 (14.3)	15 (23.8)	3 (4.8)	23 (36.5)	5 (7.9)	34 (54.0)	4 (6.3)	4 (6.3)	3 (4.8)	7 (11.1)
Laceration	430	200 (46.5)	37 (8.6)	5 (13.5)	9 (24.3)	2 (5.4)	12 (32.4)	5 (13.5)	21 (56.8)	3 (8.1)	3 (8.1)	2 (5.4)	7 (18.9)
Facial depression	49	46 (93.9)	23 (46.9)	3 (13.0)	3 (13.0)	0 (0.0)	18 (78.3)	2 (8.7)	6 (26.1)	3 (13.0)	3 (13.0)	1 (4.3)	1 (4.3)
Peri-orbital hematoma	355	199 (56.1)	38 (10.7)	7 (18.4)	11 (28.9)	3 (7.9)	16 (42.1)	5 (13.2)	21 (55.3)	3 (7.9)	3 (7.9)	3 (7.9)	2 (5.3)
Raccoon eyes	55	35 (63.6)	7 (12.7)	2 (28.6)	2 (28.6)	2 (28.6)	0 (0.0)	0 (0.0)	6 (85.7)	1 (14.3)	0 (0.0)	2 (28.6)	0 (0.0)
Epistaxis	285	194 (68.1)	49 (17.2)	5 (10.2)	8 (16.3)	4 (8.2)	17 (34.7)	5 (10.2)	34 (69.4)	4 (8.2)	4 (8.2)	2 (4.1)	2 (4.1)
Subconjunctival hemorrhage	69	52 (75.4)	10 (14.5)	2 (20.0)	3 (30.0)	2 (20.0)	4 (40.0)	3 (30.0)	6 (60.0)	1 (10.0)	1 (10.0)	1 (10.0)	1 (10.0)
Ocular movement limitation	13	12 (92.3)	4 (30.8)	1 (25.0)	2 (50.0)	1 (25.0)	3 (75.0)	1 (25.0)	2 (50.0)	0 (0.0)	0 (0.0)	0 (0.0)	0 (0.0)
Diplopia	20	13 (65.0)	5 (25.0)	1 (20.0)	1 (20.0)	0 (0.0)	4 (80.0)	0 (0.0)	0 (0.0)	0 (0.0)	0 (0.0)	0 (0.0)	0 (0.0)
Infra-orbital nerve paresthesia	62	50 (80.6)	13 (21.0)	0 (0.0)	3 (23.1)	0 (0.0)	11 (84.6)	1 (7.7)	3 (23.1)	1 (7.7)	0 (0.0)	0 (0.0)	0 (0.0)
Subjective malocclusion ^3^	47	27 (57.4)	14 (29.8)	1 (7.1)	4 (28.6)	0 (0.0)	6 (42.9)	0 (0.0)	1 (7.1)	2 (14.3)	2 (14.3)	0 (0.0)	5 (35.7)
Objective malocclusion ^3^	23	14 (60.9)	9 (39.1)	0 (0.0)	1 (11.1)	0 (0.0)	2 (22.2)	0 (0.0)	1 (11.1)	2 (22.2)	2 (22.2)	0 (0.0)	5 (55.6)
Tooth mobility or avulsion	48	33 (68.8)	17 (35.4)	1 (5.9)	2 (11.8)	1 (5.9)	1 (5.9)	1 (5.9)	6 (35.3)	1 (5.9)	4 (23.5)	1 (5.9)	9 (52.9)
Palpable step-off	68	62 (91.2)	28 (41.2)	5 (17.9)	9 (32.1)	2 (7.1)	13 (46.4)	3 (10.7)	11 (39.3)	3 (10.7)	2 (7.1)	2 (7.1)	3 (10.7)
Maxillary mobility	36	20 (55.6)	10 (27.8)	2 (20.0)	2 (20.0)	0 (0.0)	2 (20.0)	1 (10.0)	6 (60.0)	4 (40.0)	4 (40.0)	1 (10.0)	2 (20.0)

^1^ Presented as the number of patients from the total number of patients with a positive physical examination finding

^2^ Presented as the number of patients from the total number of patients diagnosed with a midfacial fracture

^3^Excluding patients with both a midfacial and mandibular trauma

### Treatment of mandibular fractures

Mandibular fractures were diagnosed in 23.6% (*n* = 66) of the patients. Symphyseal or parasymphyseal (*n* = 24), corpus (*n* = 17) and condylar process (*n* = 44) fractures were the most common mandibular fracture subtypes observed (Table 1). Regarding the patients that were diagnosed with a mandibular fracture, 29 (43.9%) were treated conservatively, 4 (6.1%) received closed treatment and 33 (50.0%) received open treatment. The treatment outcomes of the different mandibular fracture subtypes are presented in Table 1. Ramus (57.1%) and condylar process (47.7%) fractures were often treated conservatively. Open treatment was commonly observed for patients with fractures of the symphyseal or parasymphyseal area (75.0%), angle (100.0%), coronoid process (75.0%) and dentoalveolar complex (100%).

### Findings related to mandibular fractures requiring treatment

Mouth opening limitation (61.4%), palpable step-off (94.1%), tooth mobility or avulsion (61.1%), objective malocclusion (66.7%), a positive axial chin pressure test (61.0%), and positive tongue blade bite test (68.2%) were the physical examination findings most often associated with the presence of a mandibular fracture (Table 2). The physical examination findings that were commonly seen with a mandibular fracture requiring surgical treatment were palpable step-off (82.4%), tooth mobility or avulsion (44.4%), objective malocclusion (42.9%), a positive tongue blade bite test (40.3%) and a positive axial chin pressure test (40.2%). The outcomes of physical examination findings for the specific mandibular fractures subtypes requiring active treatment are presented in Table 3.

**Table 3:** Physical examination findings associated with mandibular fractures requiring treatment

Physical examination finding	Total observed	Mandibular fractures (*n* (%)) ^1^		Mandibular fractures requiring active treatment (*n* (%)) ^2^						
		Fractures identified	Fractures requiring active treatment	Symphyseal or parasymphyseal	Corpus	Angular	Ramus	Coronoid	Condylar process	Dentoalveolar complex
Swelling	105	50 (47.6)	25 (23.8)	12 (48.0)	7 (28.0)	8 (32.0)	2 (8.0)	3 (12.0)	14 (56.0)	1 (4.0)
Extra-oral laceration	101	36 (35.6)	19 (18.8)	9 (47.4)	6 (31.6)	0 (0.0)	1 (5.3)	2 (10.5)	16 (84.2)	1 (5.3)
Jaw movement pain	129	59 (45.7)	34 (26.4)	18 (52.9)	10 (29.4)	7 (20.6)	2 (5.9)	1 (2.9)	22 (64.7)	0 (0.0)
Mouth opening limitation	88	54 (61.4)	30 (34.1)	16 (53.3)	10 (33.3)	5 (16.7)	2 (6.7)	1 (3.3)	20 (66.7)	0 (0.0)
Inferior alveolar nerve paresthesia	8	4 (50.0)	3 (37.5)	1 (33.3)	1 (33.3)	1 (33.3)	0 (0.0)	0 (0.0)	2 (66.7)	0 (0.0)
Intra-oral hematoma	35	19 (54.3)	12 (34.3)	6 (50.0)	5 (41.7)	2 (16.7)	1 (8.3)	1 (8.3)	7 (58.3)	1 (8.3)
Intra-oral laceration	62	24 (38.7)	18 (29.0)	8 (44.4)	7 (38.9)	5 (27.8)	1 (5.6)	1 (5.6)	10 (55.6)	1 (5.6)
Palpable step-off	17	16 (94.1)	14 (82.4)	6 (42.9)	6 (42.9)	4 (28.6)	2 (14.3)	3 (21.4)	7 (50.0)	1 (7.1)
Tooth mobility or avulsion	18	11 (61.1)	8 (44.4)	6 (75.0)	1 (12.5)	1 (12.5)	0 (0.0)	0 (0.0)	6 (75.0)	0 (0.0)
Subjective malocclusion ^3^	69	37 (53.6)	23 (33.3)	15 (65.2)	6 (26.1)	3 (13.0)	2 (8.7)	1 (4.3)	16 (69.6)	0 (0.0)
Objective malocclusion ^3^	42	28 (66.7)	18 (42.9)	10 (55.6)	5 (27.8)	3 (16.7)	1 (5.6)	1 (5.6)	12 (66.7)	0 (0.0)
Angular compression test pain	96	51 (53.1)	33 (34.4)	17 (51.5)	10 (30.3)	7 (21.2)	2 (6.1)	1 (3.0)	21 (63.6)	0 (0.0)
Axial chin pressure pain	82	50 (61.0)	33 (40.2)	17 (51.5)	10 (30.3)	7 (21.2)	2 (6.1)	1 (3.0)	22 (66.7)	0 (0.0)
Tongue blade bite test	22	15 (68.2)	12 (54.5)	7 (58.3)	4 (33.3)	2 (16.7)	0 (0.0)	0 (0.0)	9 (75.0)	0 (0.0)

^1^Presented as the number of patients from the total number of patients with a positive physical examination finding

^2^Presented as the number of patients from the total number of patients diagnosed with a midfacial fracture

^3^Excluding patients with both a midfacial and mandibular trauma

### Diagnostic accuracy

The diagnostic accuracy of the individual physical examination findings for both the midfacial and mandibular trauma patients who needed fracture treatment is presented in Table 4. The sensitivity of the findings for midfacial trauma patients was high for swelling, and specificity was found high for almost all physical examination findings except for swelling, laceration, periorbital hematoma and epistaxis. The NPV was high for all findings. The sensitivity of the findings for mandibular trauma patients was high for jaw movement pain, the angular compression test and the axial chin pressure test. High specificity was found for inferior alveolar nerve paresthesia, intra-oral hematoma, palpable step-off, tooth mobility or avulsion, and a positive tongue blade bite test. For jaw movement pain, a NPV of 100.0 and infinitesimal LR− was found. NPV was also found high for all other findings.

**Table 4:** Accuracy of physical examination findings for midfacial and mandibular trauma patients requiring active treatment

Midface	Statistics					
	Sens. (CI)	Spec. (CI)	PPV (CI)	NPV (CI)	LR+ (CI)	LR− (CI)
Swelling	85.1 (75.3–91.5)	19.4 (16.6–22.5)	10.1 (8.0–12.8)	92.4 (86.9–95.7)	1.1 (1.0–1.2)	0.8 (0.4–1.4)
Laceration	50.0 (38.9–61.1)	43.2 (39.6–46.9)	8.6 (6.3–11.6)	89.0 (85.2–91.9)	0.9 (0.7–1.1)	1.2 (0.9–1.5)
Facial depression	33.3 (23.4–45.1)	96.1 (94.4–97.4)	46.9 (33.7–60.6)	93.4 (91.3–95.0)	8.6 (5.2–14.3)	0.7 (0.6–0.8)
Peri-orbital hematoma	51.4 (40.2–62.4)	54.2 (50.5–57.9)	10.7 (7.9–14.4)	91.2 (88.1–93.6)	1.1 (0.9–1.4)	0.9 (0.7–1.1)
Raccoon eyes	9.7 (4.8–18.7)	93.1 (90.9–94.7)	12.7 (6.3–24.0)	90.8 (88.5–92.7)	1.4 (0.7–3.0)	1.0 (0.9–1.0)
Epistaxis	68.1 (56.6–77.7)	65.4 (61.8–68.9)	17.2 (13.3–22.0)	95.1 (92.8–96.7)	2.0 (1.6–2.4)	0.5 (0.3–0.7)
Subconjunctival hemorrhage	15.2 (8.4–25.7)	91.0 (88.6–93.0)	14.5 (8.1–24.7)	91.5 (89.1–93.4)	1.7 (0.9–3.1)	0.9 (0.8–1.0)
Ocular movement limitation	6.3 (2.5–15.0)	98.6 (97.3–99.3)	30.8 (12.7–57.6)	91.3 (88.9–93.1)	4.4 (1.4–13.9)	1.0 (0.9–1.0)
Diplopia	8.1 (3.5–17.5)	97.6 (96.1–98.6)	25.0 (11.2–46.9)	91.6 (89.2–93.4)	3.4 (1.3–9.1)	0.9 (0.9–1.0)
Infra-orbital nerve paresthesia	20.0 (12.1–31.3)	92.2 (89.9–94.1)	21.0 (12.7–32.6)	91.8 (89.4–93.7)	2.6 (1.5–4.5)	0.9 (0.8–1.0)
Subjective malocclusion ^1^	12.5 (5.0–28.1)	97.2 (94.8–98.5)	30.8 (12.7–57.6)	91.8 (88.4–94.3)	4.5 (1.5–13.8)	0.9 (0.8–1.0)
Objective malocclusion ^1^	0.0 (0.0–11.0)	98.5 (96.5–99.3)	0.0 (0.0–43.4)	91.2 (87.8–93.7)	1/∞	1.0 (1.0–1.0)
Tooth mobility or avulsion	23.0 (14.9–33.7)	95.4 (93.6–96.8)	35.4 (23.4–49.6)	91.9 (89.7–93.7)	5.0 (2.9–8.7)	0.8 (0.7–0.9)
Palpable step-off	38.9 (28.5–50.4)	93.9 (91.8–95.5)	41.2 (30.3–53.0)	93.4 (91.2–95.0)	6.4 (4.2–9.7)	0.7 (0.5–0.8)
Maxillary mobility	15.4 (8.6–26.1)	96.0 (94.2–97.3)	27.8 (15.8–44.0)	91.9 (89.6–93.7)	3.9 (1.9–7.6)	0.9 (0.8–1.0)
Mandible						
Swelling	67.6 (51.5–80.4)	67.1 (60.9–72.7)	23.8 (16.7–32.8)	93.1 (88.4–96.0)	2.1 (1.5–2.7)	0.5 (0.3–0.8)
Extra-oral laceration	51.4 (35.9–66.6)	66.3 (60.1–71.9)	18.8 (12.4–27.5)	89.9 (84.7–93.5)	1.5 (1.1–2.2)	0.7 (0.5–1.0)
Jaw movement pain	100.0 (89.8–100.0)	59.9 (53.6–65.9)	26.4 (19.5–34.6)	100.0 (97.4–100.0)	2.5 (2.1–2.9)	1/∞
Mouth opening limitation	88.2 (73.4–95.3)	75.7 (69.9–80.7)	34.1 (25.0–44.5)	97.8 (94.6–99.2)	3.6 (2.8–4.7)	0.2 (0.1–0.4)
Inferior alveolar nerve paresthesia	9.1 (3.1–23.6)	97.8 (95.0–99.1)	37.5 (13.7–69.4)	88.1 (83.6–91.6)	4.1 (1.0–16.5)	0.9 (0.8–1.0)
Intra-oral hematoma	40.0 (24.6–57.7)	90.2 (85.7–93.4)	34.3 (20.8–50.8)	92.1 (87.9–95.0)	4.1 (2.3–7.3)	0.7 (0.5–0.9)
Intra-oral laceration	56.3 (39.3–71.8)	81.4 (75.9–85.8)	29.0 (19.2–41.3)	93.2 (88.9–95.9)	3.0 (2.0–4.5)	0.5 (0.4–0.8)
Palpable step-off	42.4 (27.2–59.2)	98.7 (96.3–99.6)	82.4 (59.0–93.8)	92.5 (88.5–95.1)	33.4 (10.1–110.0)	0.6 (0.4–0.8)
Tooth mobility or avulsion	22.2 (11.7–38.1)	95.8 (92.4–97.7)	44.4 (24.6–66.3)	89.1 (84.6–92.3)	5.3 (2.2–12.5)	0.8 (0.7–1.0)
Subjective malocclusion ^1^	89.5 (68.6–97.1)	64.1 (48.4–77.3)	54.8 (37.8–70.8)	92.6 (76.6–97.9)	2.5 (1.6–3.9)	0.2 (0.0–0.6)
Objective malocclusion ^1^	68.4 (46.0–84.6)	77.5 (62.5–87.7)	59.1 (38.7–76.7)	83.8 (68.9–92.3)	3.0 (1.6–5.8)	0.4 (0.2–0.8)
Angular compression test pain	97.1 (85.1–99.5)	73.0 (66.9–78.3)	34.4 (25.6–44.3)	99.4 (96.8–99.9)	3.6 (2.9–4.5)	0.0 (0.0–0.3)
Axial chin pressure pain	97.1 (85.1–99.5)	78.3 (72.5–83.2)	40.2 (30.3–51.1)	99.4 (96.9–99.9)	4.5 (3.5–5.8)	0.0 (0.0–0.3)
Tongue blade bite test	75.0 (50.5–89.8)	93.5 (88.5–96.4)	54.5 (34.7–73.1)	97.3 (93.3–98.9)	11.6 (6.0–22.4)	0.3 (0.1–0.6)

*Prev*. prevalence, *Sens*. sensitivity, *Spec*. specificity, *Pr*. pre-test probability, *PPV* positive predictive value, *NPV* negative predictive value, *LR*+ positive likelihood ratio, *LR* − negative likelihood ratio

^1^Excluding patients with both a midfacial and mandibular trauma

### Clinical decision aids

Clinical decision aids were successfully constructed to discern patients with midfacial or mandibular fractures that require treatment. For midfacial trauma patients, the clinical decision aid consisted of facial depression, epistaxis, ocular movement limitation, palpable step-off, objective malocclusion, and tooth mobility or avulsion. The aid had a sensitivity of 97.3 (90.7–99.3), a specificity of 38.6 (35.0–42.3), a NPV of 99.3 (97.3–99.8), and a LR− of 0.1 (0.0–0.3) when all the physical examination findings were observed as being absent. The decision aid helped in accurately picking out 34.9% (*n* = 267) of the patients who required active treatment for midfacial fractures. A total of 2 (0.3%) fracture patients were not identified, both of whom had nasal fractures. The clinical decision for mandibular trauma patients consisted of mouth opening limitation, jaw movement pain, objective malocclusion, and tooth mobility or avulsion, and had a sensitivity of 100.0 (90.6–100.0), a specificity of 39.1 (33.2–45.4), a NPV of 100.0 (96.1–100.0) and an infinitesimal LR−. The details of the clinical decision aids are presented in Table 5.

**Table 5:** Clinical decision aid for discerning patients with fractures that require active treatment

Clinical decision aid	Physical examination findings	Definitions	Contingency table outcomes		Cumulative diagnostic accuracy					
			TN (%)	FN (%)	Sens. (CI)	Spec. (CI)	PPV (CI)	NPV (CI)	LR+ (CI)	LR− (CI)
Midfacial trauma	Facial depression	Unilateral flattening of the malar eminence or zygomaticomaxillary complex	647 (84.5)	46 (6.0)	37.8 (27.6–49.2)	93.5 (91.4–95.1)	38.4 (28.1–49.8)	93.4 (91.3–95.0)	5.8 (3.9–8.7)	0.7 (0.6–0.8)
	Epistaxis	A unilateral or bilateral active or past nosebleed	434 (56.7)	14 (1.8)	81.1 (70.7–88.4)	62.7 (59.1–66.2)	18.9 (14.9–23.5)	96.9 (94.8–98.1)	2.2 (1.9–2.5)	0.3 (0.2–0.5)
	Ocular movement limitation	Unilateral restricted gazing or limitation of the eye movements in any direction	405 (52.9)	12 (1.6)	83.8 (73.8–90.5)	58.5 (54.8–62.1)	17.8 (14.1–22.1)	97.1 (95.0–98.3)	2.0 (1.8–2.3)	0.3 (0.2–0.5)
	Palpable step-off	The presence of a bony step-off found during palpation of the zygomatic arch, infra-orbital rim, supra and lateral orbital rim, nasal bridge and zygomaticoalveolar crest intra-orally	378 (49.3)	8 (1.0)	89.2 (80.1–94.4)	54.6 (50.9–58.3)	17.4 (13.9–21.5)	97.9 (96.0–98.9)	2.0 (1.8–2.2)	0.2 (0.1–0.4)
	Objective malocclusion	Objectively identified traumatic misalignment of the maxillary and mandibular dental arches	278 (36.3)	5 (0.7)	93.2 (85.1–97.1)	40.2 (36.6–43.9)	14.3 (11.4–17.7)	98.2 (95.9–99.2)	1.6 (1.4–1.7)	0.2 (0.1–0.4)
	Tooth mobility or avulsion	Mobility or avulsion of any maxillary tooth element	267 (34.9)	2 (0.3)	97.3 (90.7–99.3)	38.6 (35.0–42.3)	14.5 (11.7–17.9)	99.3 (97.3–99.8)	1.6 (1.5–1.7)	0.1 (0.0–0.3)
Mandible trauma	Mouth opening limitation	The reported inability to fully open the mouth or a measured mouth opening of less than 35 mm	181 (64.6)	4 (1.4)	89.2 (75.3–95.7)	74.5 (68.7–79.6)	34.7 (25.9–44.7)	97.8 (94.6–99.2)	3.5 (2.7–4.5)	0.1 (0.1–0.4)
	Jaw movement pain	Evident presence of pain during opening, protrusion or lateral movement of the mandible	137 (48.9)	0 (0.0)	100.0 (90.6–100.0)	56.4 (50.1–62.5)	25.9 (19.4–33.6)	100.0 (97.3–100.0)	2.3 (2.0–2.6)	1/∞
	Objective malocclusion	Objectively identified traumatic misalignment of the maxillary and mandibular dental arches	101 (36.1)	0 (0.0)	100.0 (90.6–100.0)	41.6 (35.5–47.8)	20.7 (15.4–27.2)	100.0 (96.3–100.0)	1.7 (1.5–1.9)	1/∞
	Tooth mobility or avulsion	Mobility or avulsion of any mandibular tooth element	95 (33.9)	0 (0.0)	100.0 (90.6–100.0)	39.1 (33.2–45.4)	20.0 (14.9–26.3)	100.0 (96.1–100.0)	1.6 (1.5–1.8)	1/∞

*TN* true negatives, *FN* false negatives, *NPV* negative predictive value, *LR* − negative likelihood ratio, *Sens*. sensitivity

## Discussion

Maxillofacial injury is frequently observed in patients admitted to the emergency department with trauma. Early recognition of maxillofacial fractures in these patients is essential, in particular because missing these fractures may lead to a decrease in esthetic and functional outcomes in the long term. Moreover, missing fractures that require surgical intervention could necessitate a secondary reconstruction, leading to additional healthcare costs, increased burden and, potentially, a poor outcome. Subjecting each maxillofacial trauma patient to a structural physical examination may help in identifying or ruling out these fractures at an early stage of treatment. In this prospective multicenter study, we assessed the diagnostic accuracy of the physical examination findings for midfacial and mandibular fractures requiring active treatment. Clinical decision aids were constructed focusing on ruling out patients with these type of fractures, resulting in a NPV of 99.3% for midfacial trauma patients, and a NPV of 100.0% for mandibular trauma patients. When all the related physical examination findings in these clinical decision aids are absent means one can successfully rule out patients with midfacial and mandibular fractures requiring treatment.

Our study identified how individual physical examination findings are associated with different subtypes of midfacial and mandibular fractures that require active treatment. For example, midfacial fractures were found in almost every patient with facial depression and almost 50% of the treated fractures were associated with fractures of the zygomaticomaxillary complex. Another example is that mandibular fractures were frequently found in patients with malocclusion, most of whom had to be treated. Most of these patients presented with symphyseal, parasymphyseal and condylar process subtype fractures, supporting the fact that displacement of fractures in these regions cause traumatic misalignment of the dental arches. Specific physical examination findings can be highly effective in the diagnosis of maxillofacial fracture subtypes requiring treatment in emergency department patients, and radiological imaging should, therefore, be strongly considered for them. Understanding these individual physical examination findings can be useful for early identification of a patient at risk of midfacial or mandibular fractures.

In this study, we found that the sensitivity remained low for physical examination findings related to midfacial trauma whereas the sensitivity of the findings for mandibular trauma patients was high for jaw movement pain, a positive angular compression test and a positive axial chin pressure chin test. The specificity was high for most of the midfacial and mandibular physical examination findings, indicating that these findings are commonly absent among patients whose fractures can be treated conservatively or do not have a fracture. This is supported by the fact that almost all the physical examination findings produced an exceptionally high NPV and, contrarily, a low PPV. Our results suggest that the absence of these physical examination findings means the unlikelihood of a midfacial or mandibular fracture that requires treatment. Therefore, these individual findings can be used to stratify patients into low or high-risk fracture groups.

Although individual physical examination findings can be useful, it is of particular interest how a combination of findings can perform as a clinical decision aid in the emergency department. In our study, clinical decision aids were constructed with the aim to differentiate patients without or with midfacial or mandibular fractures that require active treatment (e.g., closed or surgical treatment). An approach was chosen in which the physicians assessed the physical examination findings not knowing the outcome of interest, representing a blinded clinical workflow of assessing emergency department patients. The clinical decision aid constructed for midfacial trauma patients consisted of facial depression, epistaxis, ocular movement limitation, palpable step-off, malocclusion, and tooth mobility or avulsion. The absence of all these findings produced a sensitivity of 97.3%, a specificity of 38.6, and a NPV of 99.3%. The clinical decision aid only misdiagnosed two patients (i.e., false negatives) with nasal fractures that required a closed treatment protocol. These two patients were missed despite including palpation of the nasal bridge and epistaxis in the nasal related physical examination findings. Nasal fractures are commonly found in maxillofacial trauma patients, emphasizing the need to consider these fractures for each patient suffering any maxillofacial trauma. Moreover, because the nose projects from the face, any nasal fracture displacements may have important esthetic consequences. Nevertheless, the clinical decision aid accurately picked out the majority of patients with midfacial fractures that required active treatment. Previous research is limited and preliminary focused on the diagnosis of orbital fractures requiring treatment. The authors of a prospective cohort study of 2262 emergency department patients with blunt orbital trauma constructed an orbital fracture risk score focusing on the need for emergent surgical intervention [5]. One point was assigned for: orbital rim tenderness, periorbital emphysema, subconjunctival hemorrhage, impaired extra-ocular movement, painful extra-ocular movement and epistaxis. The authors stated the risk score was successful as only three patients had been misdiagnosed. In another retrospective cohort study of 912 orbital trauma patients, an orbital fracture risk score was constructed to predict the need for surgery [11]. One point was given for periorbital emphysema and male sex, and two points for diplopia and infra-orbital nerve paresthesia. A cutoff of two points was defined as the best compromise for the risk of surgical intervention, producing a NPV of 92.1% and a sensitivity of 82.5%.

The clinical decision aid we constructed for mandibular trauma patients consisted of mouth opening limitation, jaw movement pain, malocclusion, tooth mobility or avulsion. The clinical decision aid correctly discerned all the patients who did not require active treatment for mandibular fractures through the absence of the physical examination findings. To the best of our knowledge, no studies have been conducted focusing on such a clinical decision aid. Our clinical decision aids were only constructed with physical examination findings. The main advantage is that the decisions are consistent, also for patients with unclear or unverifiable components such as age, sex or mechanism of injury. The clinical decision aids allow for early bedside management during an early stage of the primary or secondary assessment. Maxillofacial trauma patients often have concomitant injuries and our clinical decision aids might help in stratifying which injuries require prioritization for resuscitation. Moreover, the clinical decision aids can be used to identify the patients requiring a consultation with an oral and maxillofacial surgeon or an otolaryngologist.

Our study has several limitations. First, only those patients who had undergone radiological imaging were included. Nevertheless, we focused on patients who required active treatment and one would expect those not needing radiological imaging as having low fracture risks. Second, the physical examination findings were assessed by emergency physicians with varying years of experience. In addition, these physician are less exposed to maxillofacial trauma patients compared to fully trained oral and maxillofacial surgeons that are more frequently faced with patients that require treatment. Nevertheless, we sought to standardize the physician examination using a tripartite strategy to ensure that testing of the physical examination findings was conducted similar for each patient despite the profession of the assessor. Third, the treatment decision was established using Dutch treatment protocols. However, other international treatment protocols for maxillofacial and mandibular fractures might be different, which confines the generalizability of the clinical decision aids. Thus, these clinical decision aids should be validated by future research with a new population of patients.

In conclusion, the physical examination findings in the clinical decision aids focusing on patients with midfacial or mandibular fractures that require active treatment are diagnostically accurate The clinical decision aids can successfully rule out emergency department patients with midfacial or mandibular fractures that require active treatment and so may be useful in preventing unnecessary radiological procedures.

## Supplementary Information

Below is the link to the electronic supplementary material.

Supplementary file1 Diagnostic accuracy of the clinical decision aids and physical examination findings (XLSX 273 KB)

## Data Availability

Included as supplementary material.

## Declarations

### Standards of reporting

The study was reported according to the STARD guidelines (Standards for Reporting of Diagnostic Accuracy Studies) and Methodologic Standards for Interpreting Clinical Decision Rules in Emergency Medicine [12, 13].

## References

[1] Tuckett JW, Lynham A, Lee GA, Perry M, Harrington U (2014). Maxillofacial trauma in the emergency department: a review. Surgeon.

[2] Perry M (2008). Advanced trauma life support (ATLS) and facial trauma: can one size fit all? Part 1: dilemmas in the management of the multiply injured patient with coexisting facial injuries. Int J Oral Maxillofac Surg.

[3] Allison JR, Kearns A, Banks RJ (2019). Predicting orbital fractures in head injury: a preliminary study of clinical findings. Emerg Radiol.

[4] Sitzman TJ, Hanson SE, Alsheik NH, Gentry LR, Doyle JF, Gutowski KA (2011). Clinical criteria for obtaining maxillofacial computed tomographic scans in trauma patients. Plast Reconstr Surg.

[5] Yadav K, Cowan E, Haukoos JS, Ashwell Z, Nguyen V, Gennis P (2012). Derivation of a clinical risk score for traumatic orbital fracture. J Trauma Acute Care Surg.

[6] Sitzman TJ, Sillah NM, Hanson SE, Gentry LR, Doyle JF, Gutowski KA (2015). Validation of clinical criteria for obtaining maxillofacial computed tomography in patients with trauma. J Craniofac Surg.

[7] Holmgren EP, Dierks EJ, Homer LD, Potter BE (2004). Facial computed tomography use in trauma patients who require a head computed tomogram. J Oral Maxillofac Surg.

[8] Exadaktylos AK, Sclabas GM, Smolka K, Rahal A, Andres RH, Zimmermann H (2005). The value of computed tomographic scanning in the diagnosis and management of orbital fractures associated with head trauma: a prospective, consecutive study at a level I trauma center. J Trauma.

[9] Büttner M, Schlittler FL, Michel C, Exadaktylos AK, Iizuka T (2014). Is a black eye a useful sign of facial fractures in patients with minor head injuries? A retrospective analysis in a level I trauma centre over 10 years. Br J Oral Maxillofac Surg.

[10] Timashpolsky A, Dagum AB, Sayeed SM, Romeiser JL, Rosenfeld EA, Conkling N (2016). A prospective analysis of physical examination findings in the diagnosis of facial fractures: determining predictive value. Plast Surg (Oakville, Ont).

[11] Scolozzi P, Jacquier P, Courvoisier DS (2017). Can clinical findings predict orbital fractures and treatment decisions in patients with orbital trauma? Derivation of a simple clinical model. J Craniofac Surg.

[12] Huang L-K, Wang HH, Tu H-F, Fu C-Y (2017). Simultaneous head and facial computed tomography scans for assessing facial fractures in patients with traumatic brain injury. Injury.

[13] Harrington AW, Pei KY, Assi R, Davis KA (2018). External validation of University of Wisconsin’s clinical criteria for obtaining maxillofacial computed tomography in trauma. J Craniofac Surg.

[14] Bossuyt PM, Reitsma JB, Bruns DE, Bruns DE, Glasziou PP, Irwig L (2015). STARD 2015: an updated list of essential items for reporting diagnostic accuracy studies1. Radiology.

[15] Green SM, Schriger DL, Yealy DM (2014). Methodologic standards for interpreting clinical decision rules in emergency medicine: 2014 update. Ann Emerg Med.

